# A case of recurrence and metastasis of pheochromocytoma: a case report

**DOI:** 10.3389/fcvm.2026.1883194

**Published:** 2026-07-28

**Authors:** Xiaofang Zhang, Qiongying Wang, Jing Yu

**Affiliations:** Department of Cardiology, The Second Hospital & Clinical Medical School, Lanzhou University, Lanzhou, China

**Keywords:** adrenal gland, chemotherapy, pheochromocytoma, secondary hypertension, tumor recurrence

## Abstract

Pheochromocytomas are rare tumors that develop from chromaffin cells of the adrenal medulla or from extra-adrenal paraganglia . These tumors frequently produce catecholamines, and excessive secretion can lead to a variety of symptoms, including episodic hypertension and metabolic disorders. Such complications can significantly impact cardiovascular health as well as kidney and vascular function, and there is notable concern regarding recurrence and metastasis following surgical resection. This article analyzes the case characteristics, diagnosis, and treatment of a patient with pheochromocytoma who experienced relapse after surgical resection, thereby providing a reference for clinical practice.

## Introduction

Pheochromocytomas and paragangliomas (PPGLs) are neuroendocrine tumors that originate from the catecholamine-producing adrenal medulla or extra-adrenal paraganglia, with an annual incidence of only 0.2–0.8 per 100,000 individuals (1). Surgical resection remains the treatment of choice and is curative in most cases; however, recurrence may occur due to various factors, including metastatic disease, multifocal tumors, hereditary syndromes, and incomplete resection (2).

## Case presentation

The patient, a 40-year-old woman of Han ethnicity, was admitted to our hospital in January 2021, presenting with the chief complaint of “episodic headache accompanied by elevated blood pressure for over 8 years, with a recurrence in the past 3 years.” Eight years prior to admission (2013), the patient began experiencing symptoms consistent with chromaffin cell tumors, including episodic headaches, dizziness, neck pain, palpitations, nausea, vomiting, and episodes of hypertension, with blood pressure surging to 180/101 mmHg, although the symptoms subsided spontaneously. Medical attention was sought at a local hospital, where an abdominal ultrasound suggested “a left adrenal tumor, possibly a pheochromocytoma.” Following further urological evaluations, a diagnosis of “left adrenal space-occupying lesion” was made, and laparoscopic resection was performed. The tumor, measuring approximately 50 mm × 60 mm, was completely excised. Pathological examination indicated a high likelihood of malignant pheochromocytoma (PCC), with Ki-67 > 1% and chromogranin A (CgA) (+++). Postoperatively, the patient did not report the aforementioned symptoms and did not require antihypertensive medication, with blood pressure monitoring showing fluctuations of “110–130/70–80 mmHg.” Three years before this admission (2018), the patient experienced a recurrence of symptoms, which presented more severely and were accompanied by dyspnea and chest discomfort. Blood pressure readings reached “180–190/100–110 mmHg,” occasionally rendering measurements impossible due to excessive elevation. Symptoms gradually alleviated with rest, with normal blood pressure averaging around “120/80 mmHg” during non-episodic periods. An adrenal contrast-enhanced CT scan revealed no evidence of recurrence or metastasis. The patient was prescribed “benazepril hydrochloride 10 mg qd” for antihypertensive management. Following this, intermittent recurrence of symptoms occurred without a clear pattern, and the patient occasionally discontinued the medication and eventually returned to our hospital for follow-up. Outpatient management labeled the case as “secondary hypertension,” leading to hospital admission.

Regarding her history, elevated fasting blood glucose was noted during a 2019 physical examination, which prompted the initiation of intermittent treatment with “metformin sustained-release tablets 0.5 g bid,” although glycemic control status remains unknown. Her surgical history includes a left adrenal pheochromocytoma resection, which was performed in 2013. In terms of her menstrual and reproductive history, menarche occurred at 14 years, her last menstrual period recorded on 24 January 2021, and she reported regular menstrual cycles. The patient was married at 28 years and has one healthy son. Her family history reveals that both parents are healthy, with no known family history of genetic diseases.

Routine blood and urine tests revealed no significant abnormalities. A complete biochemical analysis of the renin–angiotensin series (standing position) indicated an aldosterone level of 8.94 ng/dL, a plasma renin activity of 2.76 ng/mL/h, and an aldosterone-to-renin ratio of 3.23. On the second day of hospitalization at 5:00 a.m., the patient experienced recurrent headache and palpitations, with a blood pressure reading of 189/100 mmHg and a pulse rate of 93 bpm. These symptoms resolved spontaneously after approximately 10 min, after which her blood pressure decreased to 133/87 mmHg. Blood and urine catecholamine tests were immediately performed, the results of which are provided in Tables 1–4. Whole-exome sequencing identified a mutation in fumarate hydratase (*FH*) gene, specifically NM_000143-exon 10:c.1431_1433dupAAA (p.K477dup), which is classified as “uncertain significance” according to the ACMG guidelines.

**Table 1:** Plasma catecholamines (supine position).

Component	Value (pmol/L)	Reference range (pmol/L)
Dopamine (DA)	71.20	Supine or standing ≤ 195.7
Epinephrine (E)	2,824.20	Supine ≤ 605.4; standing ≤ 769
Norepinephrine (NE)	2,797.20	Supine 414.0–4,435.5; standing 1,182.8–10,054

**Table 2:** Plasma methoxyadrenalin (three items).

Component	Value (nmol/L)	Reference range (nmol/L)
3-Methoxytryptamine	<0.08	<0.18
Methoxyadrenalin	12.6	≤0.50
Methoxynorepinephrine	13.5	≤0.90

**Table 3:** Twenty-four-hour urinary catecholamines.^a^

Component	Value (mmol/24 h)	Reference range (mmol/24 h)
Epinephrine (E)	1,186.32	4.31–61.6
Norepinephrine (NE)	1,411.43	60.0–352
Dopamine (DA)	2,043.93	750.00–2,088.00 (for women)

a Twenty-four-hour urine volume: 2,200 mL.

**Table 4:** Twenty-four-hour urinary free catecholamine data.^a^

Component	Value (mmol/24 h)	Reference range (mmol/h)
Catecholamines	7,182.00	216
Norepinephrine	4,723.00	312
3-Methoxytryptamine	229.00	<382

a Twenty-four -hour urine volume: 2,200 mL.

**Figure 1:**
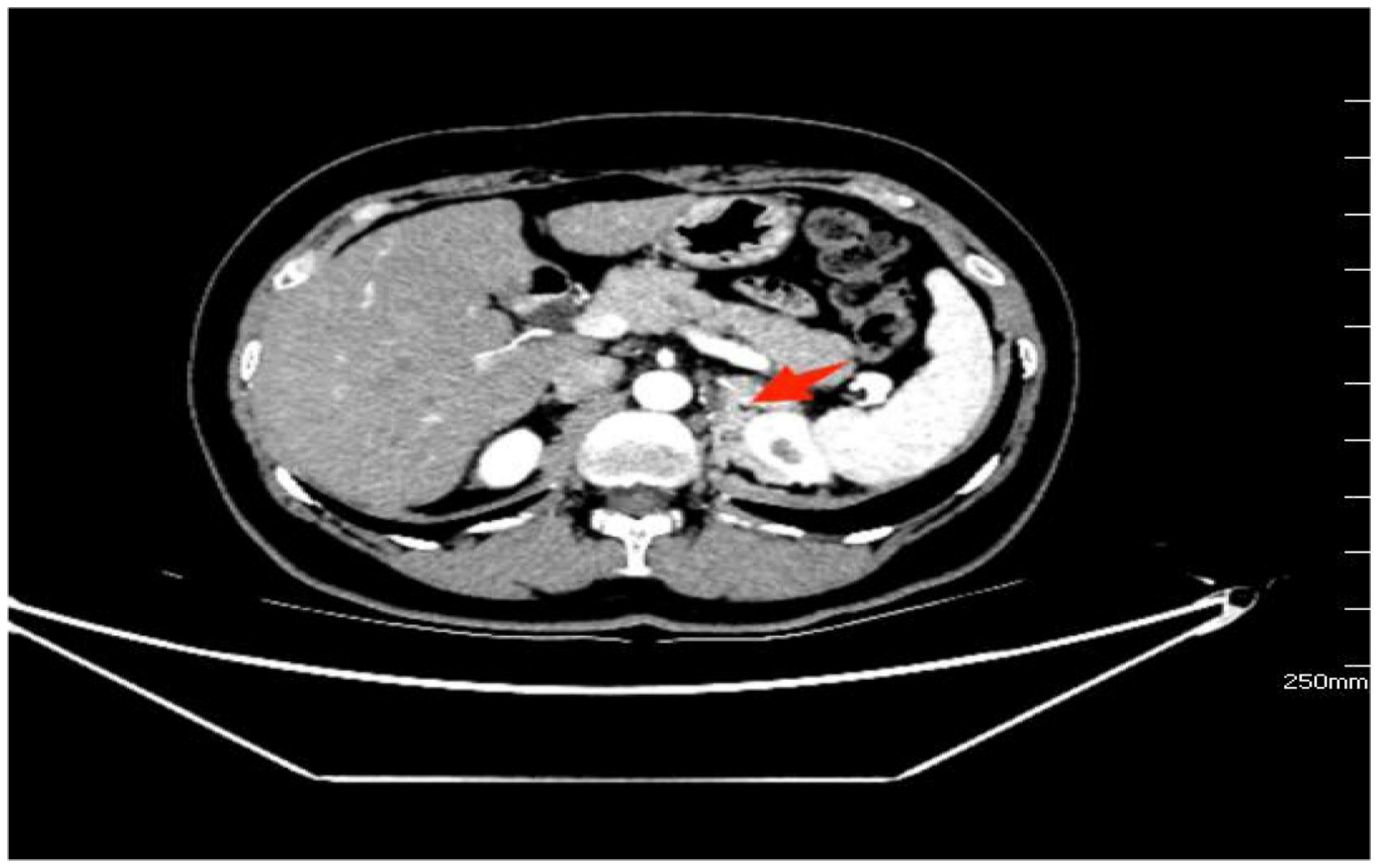
Enhanced abdominal CT scan in January 2021 showing the left adrenal gland.

The electrocardiogram and echocardiogram revealed no significant abnormalities. Concurrently, contrast-enhanced CT scans (including renal, renal vascular, and adrenal assessments) and whole-body positron emission tomography (PET)/CT tomography were performed, both of which showed positive findings suggestive of a recurrence of pheochromocytoma. These results are summarized in Table 5, and the imaging of the adrenal changes is depicted in Figure 1.

**Table 5:** Medical imaging result comparison.

Imaging type	Findings	Diagnostic suggestions
Contrast-enhanced CT (renal, renal vascular, adrenal)	Postresection of left adrenal pheochromocytoma, multiple nodular and patchy areas of abnormal density were observed in the left adrenal region, hepato-gastric space, and perirenal area, suggestive of tumor metastasis (Figure 1)	These findings are consistent with the imaging of adrenal metastases and pheochromocytomas, as described in the literature
PET/CT (whole-body tomography)	Soft tissue shadow in the left adrenal resection area, partially extending anterior to the diaphragm around the abdominal aorta to the contralateral side, extending downward to the left diaphragmatic pedicle, lumbar quadratus muscle surface, and adjacent local peritoneum. A soft tissue nodule is observed in the right abdominal wall fat layer near the medial border of the external oblique muscle at the umbilical level. Small lymph nodes adjacent to the left cardia are present	Differential diagnosis includes “tumor recurrence with spread to the aforementioned sites and lymph node metastasis” vs. “postoperative inflammatory hyperplastic changes”

Leveraging insights from the imaging studies, and in consultation with Fuwai Hospital, our interdisciplinary team reached a consensus on the treatment approach. Because the patient has suspected recurrent pheochromocytoma, characterized by multiple hypermetabolic nodules, alongside symptoms such as hypertension, heart palpitations, and excessive sweating, she is not considered a candidate for surgical intervention. Recommended chemotherapy options for this patient include the cyclophosphamide, vincristine, dacarbazine (CVD) regimen or the EP regimen (etoposide and cisplatin). In addition, radiotherapy may be considered as part of the treatment plan, particularly in cases where surgery is not feasible or malignancy is present.

Due to the inability of our hospital to perform interstitial iodobenzylguanidine therapy at that time, the patient was referred to Peking Union Medical College Hospital following coordination with our department. Subsequently, a ^131^I-MIBG scintigraphy emission computed tomography (ECT) scan was performed, revealing multiple abnormal findings in the left adrenal region and left abdominal aorta that were suspicious for recurrent pheochromocytoma. In light of the inability to perform surgical resection, the patient was treated with temozolomide at a dosage of 150–200 mg/m^2^ per day for 5 consecutive days, followed by a 23-day rest period, constituting a 28-day treatment cycle. Following this regimen, the frequency of symptom episodes significantly decreased. A follow-up contrast-enhanced adrenal CT in December 2021 demonstrated a soft tissue density nodule in the right abdominal wall measuring 5 mm × 50 mm and approximately 8.5 mm in length, which was slightly smaller than previously reported.

## Discussion

Pheochromocytoma, a rare tumor of the endocrine system, is primarily treated with surgical resection. However, recurrence may occur in certain patients following surgical removal. Previous literature has identified genetic predisposition, embryonic development abnormalities, hormonal imbalances, and environmental factors as independent risk factors for the development and recurrence of pheochromocytoma (1). Case reports suggest that recurrence is typically associated with intraoperative tumor spillage or capsular rupture (2). Gao Yinjie et al. observed that patients diagnosed with PPGLs are at risk of postoperative recurrence or metastasis. Their study suggested that individuals experiencing postoperative recurrence or distant metastasis often present with an earlier age of onset, prolonged disease duration, increased tumor size, and a higher incidence of multiple lesions (3). Some studies have indicated that PPGL patients with tumors larger than 50 mm, particularly those exhibiting liquefactive necrosis, a high Ki-67 index, and strong positivity for CgA, are at a greater risk of recurrence and metastasis (4). Furthermore, patients diagnosed with PPGLs carry a higher risk of recurrence and metastasis than those with pheochromocytoma, leading to a poorer prognosis, as evidenced by recent studies and clinical reviews (5, 6). This case, characterized by a relatively young age at presentation, an intraoperative tumor size exceeding 50 mm, and a Ki-67 index greater than 1%, aligns with a high-risk profile for recurrence, as supported by prior findings and the elevated Ki-67 index.

Recurrence in this patient was definitively diagnosed through imaging 8 years after the resection of pheochromocytoma. However, symptoms consistent with pheochromocytoma excess (e.g., hypertension, palpitations, and headaches) had already emerged 5 years postsurgery. At that time, an abdominal CT scan revealed no abnormalities, presumably because the lesion was too small to be detected. The absence of regular follow-up may have hindered earlier identification of the recurrent lesion. This case highlights the critical need for rigorous, standardized surveillance of all patients following pheochromocytoma resection. In particular, for individuals who present with suggestive symptoms but negative conventional imaging, more sensitive functional imaging modalities—such as metaiodobenzylguanidine (MIBG) scintigraphy—or intensified follow-up strategies should be employed to facilitate early detection.

Regarding the timing of postoperative recurrence, a retrospective Spanish study involving 303 patients with PCC or PPGL, with a median follow-up of 4.8 years (range, 1–19 years), reported a median time from initial PCC or PPGL diagnosis to recurrence of 11.2 months (range, 0.5–174 months). Recurrent cases were evenly distributed throughout the follow-up period, indicating that PCC recurrence can occur at any time after the primary diagnosis (7). Indeed, cases of recurrence as late as 40 years postsurgery have been documented in the literature (2). These observations collectively reinforce the principle that surveillance after pheochromocytoma resection constitutes a lifelong obligation.

The patient was definitively diagnosed with pheochromocytoma, which recurred after surgical resection, resulting in no opportunities for further surgical intervention. For patients with unresectable recurrent pheochromocytoma, treatment options include ^131^I-mediated radioactive iodine therapy, radiotherapy, and chemotherapy. However, these treatments primarily serve to control tumor growth and suppress hormone levels (1). The CVD regimen, consisting of cyclophosphamide, vincristine, and dacarbazine, is recommended as a first-line chemotherapy strategy for recurrent pheochromocytoma, supported by both domestic and international literature. In addition, temozolomide monotherapy has demonstrated significant benefits, showing favorable efficacy in treating metastatic neuroendocrine tumors with a 2-year survival rate of 61% (8). Reports from international literature indicate that both combination therapy and monotherapy with temozolomide effectively reduce catecholamine release and inhibit tumor growth, offering a favorable safety profile with fewer adverse reactions (9–11). Furthermore, temozolomide is often preferred due to its oral (or intravenous) administration over a 5-day period each month, allowing patients to maintain a relatively normal routine during the remaining weeks of the treatment cycle (12). In this case, the patient was able to take temozolomide regularly and consistently, with follow-up liver function tests showing no impairment. Imaging performed 1 year after initiation of temozolomide treatment revealed no increase in tumor volume, and blood pressure remained stable under the regimen of phentolamine and metoprolol succinate, further confirming the efficacy of this therapeutic approach.

## Conclusion

While this case may not be particularly complex, it highlights the importance of recognizing that a definitive diagnosis and successful surgical resection are merely the initial steps in managing patients with pheochromocytoma. Greater emphasis must be placed on long-term follow-up and comprehensive evaluation, especially for those at high risk of recurrence or those presenting with recurrent symptoms. Moreover, the chemotherapy regimen utilized in this case provides valuable reference points for clinical practice regarding the management of inoperable recurrent lesions.

## Data Availability

The raw data supporting the conclusions of this article will be made available by the authors, without undue reservation.
